# Chromosomal integration vectors allowing flexible expression of foreign genes in *Campylobacter jejuni*

**DOI:** 10.1186/s12866-015-0559-5

**Published:** 2015-10-24

**Authors:** Adrian J. Jervis, Jonathan A. Butler, Brendan W. Wren, Dennis Linton

**Affiliations:** Faculty of Life Sciences, University of Manchester, Michael Smith Building, Manchester, M13 9PT UK; Pathogen Molecular Biology Unit, London School of Hygiene and Tropical Medicine, London, WC1E 7HT UK

**Keywords:** Campylobacter, Complementation, Expression, Promoter, Green fluorescent protein

## Abstract

**Background:**

*Campylobacter jejuni* is a major cause of human gastroenteritis yet there is limited knowledge of how disease is caused. Molecular genetic approaches are vital for research into the virulence mechanisms of this important pathogen. Vectors that allow expression of genes in *C. jejuni* via recombination onto the chromosome are particularly useful for genetic complementation of insertional knockout mutants and more generally for expression of genes in particular *C. jejuni* host backgrounds.

**Methods:**

A series of three vectors that allow integration of genes onto the *C. jejuni* chromosome were constructed by standard cloning techniques with expression driven from three different strong promoters. Following integration onto the *C. jejuni* chromosome expression levels were quantified by fluorescence measurements and cells visualized by fluorescence microscopy.

**Results:**

We have created plasmid, pCJC1, designed for recombination-mediated delivery of genes onto the *C. jejuni* chromosome. This plasmid contains a chloramphenicol resistance cassette (*cat*) with upstream and downstream restriction sites, flanked by regions of the *C. jejuni* pseudogene Cj0223. Cloning of genes immediately upstream or downstream of the *cat* gene allows their subsequent introduction onto the *C. jejuni* chromosome within the pseudogene. Gene expression can be driven from the native gene promoter if included, or alternatively from the *cat* promoter if the gene is cloned downstream of, and in the same transcriptional orientation as *cat*. To provide increased and variable expression of genes from the *C. jejuni* chromosome we modified pCJC1 through incorporation of three relatively strong promoters from the *porA*, *ureI* and *flaA* genes of *C. jejuni*, *Helicobacter pylori* and *Helicobacter pullorum* respectively. These promoters along with their associated ribosome binding sites were cloned upstream of the *cat* gene on pCJC1 to create plasmids pCJC2, pCJC3 and pCJC4. To test their effectiveness, a green fluorescent protein (*gfp*) reporter gene was inserted downstream of each of the three promoters and following integration of promoter-gene fusions onto the *C. jejuni* host chromosome, expression levels were quantified. Expression from the *porA* promoter produced the highest fluorescence, from *flaA* intermediate levels and from *ureI* the lowest. Expression of *gfp* from the *porA* promoter enabled visualization by fluorescent microscopy of intracellular *C. jejuni* cells following invasion of HeLa cells.

**Conclusions:**

The plasmids constructed allow stable chromosomal expression of genes in *C. jejuni* and, depending on the promoter used, different expression levels were obtained making these plasmids useful tools for genetic complementation and high level expression.

**Electronic supplementary material:**

The online version of this article (doi:10.1186/s12866-015-0559-5) contains supplementary material, which is available to authorized users.

## Background

Campylobacters, predominantly *Campylobacter jejuni* and *Campylobacter coli*, are the most commonly reported causal agents of zoonotic infection with an estimated 400–500 million cases annually worldwide [1]. Campylobacteriosis is an acute, generally self-limiting, diarrhoeal disease [2, 3] with a number of infrequent but serious sequelae of infection including the peripheral neuropathy Guillain-Barre syndrome. Due to its importance as a human pathogen there are many research groups employing molecular genetic approaches to investigate *C. jejuni* virulence and transmission. However, the availability of tractable genetic tools is relatively limited and our knowledge of how *C. jejuni* causes disease is thus also limited compared to other enteropathogens such as *Escherichia coli* and *Salmonella* species.

One of the key methods for investigating *C. jejuni* gene function is inactivation of specific genes through insertion of antibiotic (generally kanamycin or chloramphenicol) resistance cassettes via recombination-mediated allelic replacement [4, 5]. To confirm associated phenotypes such mutants are often genetically complemented with at least partial restoration of phenotype confirming the specific role of the gene product under investigation. The two approaches for complementation in *C. jejuni* are reintroduction of a functional gene either on a replicating plasmid [6, 7] or through chromosomal integration [8]. The latter method involves construction of suicide plasmids that allow recombination-driven introduction of functional genes at specific loci on the *C. jejuni* chromosome. These loci are chosen so as to minimise potential for unwanted effects of insertion and include intergenic region of rRNA genes [8], or pseudogenes such as Cj0046 [9, 10] and Cj0223 [11].

An important consideration when designing genetic complementation strategies is the nature of the promoter driving gene expression. Ideally one would use the promoter region from which the gene is expressed [10], but these are often difficult to identify accurately and if the gene is part of an operon, may be located some distance away potentially requiring further cloning steps. A simpler and more commonly used strategy is to use the promoter associated with the antibiotic resistance cassette and insert the gene immediately downstream without an intervening transcriptional terminator. However other more or less well characterized promoters from *C. jejuni* such as that of the iron induced gene *fdxA* have also been employed [12].

The aim of this study was to generate plasmids for integrating selected genes onto the *C. jejuni* chromosome that allow expression at different levels. To this end we employed one of three distinct *Campylobacter* or *Helicobacter* promoter regions to drive gene expression. Using these plasmids to introduce reporter gene *gfp* onto the *C. jejuni* NCTC 11168 chromosome we measured three significantly different levels of gene expression. The highest of these enabled us for the first time to readily visualize intracellular fluorescent *C. jejuni* cells expressing chromosomal *gfp* during in vitro cell invasion experiments using a standard fluorescent microscope. The use of these plasmids to generate stable highly fluorescent *C. jejuni* strains should have widespread applications and improve our knowledge of the virulence and transmission of this major pathogen. These plasmids will also be useful for both genetic complementation and more generally for expressing genes of other origin in *C. jejuni* backgrounds.

## Methods

### Bacterial strains

*Escherichia coli* XL10 gold strains (Stratagene) were grown in Luria-Bertani (LB) broth or on LB agar plates. *C. jejuni* NCTC 11168 and *H. pullorum* NCTC 12824 strains from the UK National Collection of Type Cultures were grown on Columbia agar containing 5 % defibrinated horse blood (TCS Biosciences) at 42 °C in a modified atmosphere (85 % N_2_, 10 % CO_2_, and 5 % O_2_) generated with a VA500 workstation (Don Whitley Ltd.). Chloramphenicol was used at a concentration of 17 μg/ml for *E. coli* and 34 μg/ml for *Campylobacter*. Ampicillin was used at a concentration of 100 μg/ml.

### Construction of *C. jejuni* expression vectors

The promoter regions of the *C. jejuni* NCTC 11168 *porA*, *H. pullorum* NCTC 12824 *flaA* and *H. pylori* 26695 *ureI*, genes were amplified with primer pairs porAXhoI-F/R, flaAXhoI-F/R and ureIXhoI-F/R respectively (Table 1), and cloned into the XhoI site immediately upstream of the *cat* cassette of pCJC1. A variant of the highly fluorescent *gfp* + gene [13] known as *gfp*^TCD^ [14] was amplified using primer pair gfp-F/R (Table 1) and cloned into the NdeI site created in the promoter regions. The codon-optimised *gfp*^*Cj*^ based on *gfp*^TCD^ was synthesized (Eurofins) with flanking NdeI sites.

**Table 1:** Primers used in this study

Primer	Sequence^*a*^ (5′- > 3′)
porAXhoI-F	CAA GAA CTC GAG CTT AAA ATT ACA CGC CTA GC
porAXhoI-R	AAT TCA CTC GAG CAT ATG AAT TCT CCT TGT CAA AAA TTA
flaAXhoI-F	CAA GAA AAG CTT GCT ATC AAA AAT TAA AAT GAT TGT C
flaAXhoI-R	AAT TCA AAG CTT CAT ATG AAA CTC CTT TAT ATT GCC TC
ureIXhoI-F	CAA GAA AAG CTT CCT TAA ATC CTT AGT TTT TAG C
ureIXhoI-R	AAT TCA AAG CTT CAT ATG CTT TTC CTT CCA AAC AAA AAT T
gfp-F	AGA ACC CAT ATG AGC AAA GGC GAA GAG CTG
gfp-R	AAA CTC CAT ATG TTA CTT ATA CAG TTC ATC CAT ACC

^*a*^Restriction sites underlined

### Transformation of *C. jejuni*

Electrocompetent *C. jejuni* cells were prepared and transformed with plasmid DNA using standard protocols [15].

### GFP fluorescence monitoring of *Campylobacter* cultures

Cultures of *C. jejuni* were grown on blood agar for 24 h, resuspended in Heart Infusion (HI) broth and used to inoculate 2 ml HI broth supplemented with 5 % bovine foetal serum (BFS) to an OD_600_ of 0.05. Cultures were grown in a 6-well tissue culture dish in a modified atmosphere (as above) for approximately 16 h at 42 °C with shaking at 125 rpm. When cultures reached mid-log phase (between OD_600_ 0.2 and 0.4) they were harvested by centrifugation, washed in PBS and resuspended to an OD_600_ of 1.0. A 10-fold dilution series of the bacterial suspension was made and 180 μl added in triplicate to a black-walled 96-well plate with transparent base. Both OD_600_ and fluorescence (excitation at 485/20 nm and emission at 528/20 nm) were measured on a Bio-Tek Synergy HT plate reader. Fluorescence in arbitrary units (AU) was calculated by dividing the Relative Fluorescence Units (RFU) by the corresponding OD_600_ value.

### Immunodetection of recombinant proteins

*C. jejuni* strains expressing GFP were grown as for fluorescence measurement described above and whole cell lysates prepared after normalizing by OD_600_. Western blots were performed using mouse anti-GFP antibody (Sigma) and goat anti-mouse secondary antibody (Li-Cor).

### Preparation of *C. jejuni* for fluorescence microscopy

*C. jejuni* strains expressing GFP were grown as above and approximately 10^8^ cells were washed in 1 ml of phosphate buffered saline (PBS) and resuspended in 50 μl of PBS. Approximately 10 μl of this suspension was spread on a glass slide, dried, heat fixed and 50 μl of 1 μg/ml 4′,6-diamidino-2-phenylindole (DAPI) added. Following incubation at room temperature for 20 min, slides were washed three times in PBS. Glass coverslips were adhered using Mowiol 4–88 and left to dry before imaging (see below).

### HeLa cell infection by *C. jejuni*

HeLa cells cultured in Dulbecco’s Modified Eagles Medium (DMEM) were seeded into 6-well plates containing glass coverslips at 1.5 x 10^5^ cells per well, and incubated for 24 h at 37 °C with 5 % CO_2_, to obtain a final density of 5 x 10^5^ cells per well. Cells were washed with Dulbecco’s Phosphate Buffered Saline (PBSD) twice before infection. Mid-log cultures of *C. jejuni* grown in HI broth supplemented with 5 % BFS were harvested, washed twice in 1 ml PBS and added to HeLa cell cultures at a multiplicity of infection (MOI) of 10. Plates were incubated for a further 2 h, washed three times with PBSD and serum-free medium containing 50 μg/ml gentamicin added. After 1 h cover slips were removed, incubated in PBS containing 3 % paraformaldehyde for 20 min at room temperature and washed three times in PBS, the last wash containing 10 mM glycine (pH 8.5). Coverslips were then incubated in PBS containing 0.1 % Triton X-100 for 4 min at room temperature, washed three times in PBS, stained with DAPI as above and with the fluorescent actin stain phalloidin-Atto590 (Sigma) at 1:400 dilution followed by three final PBS washes.

### Fluorescence microscopy of *C. jejuni* and HeLa cells

Images were collected on an Olympus BX51 upright microscope using a 40x/ 0.75 Plan Fln or 60x/ 0.65-1.25 Plan Fln objective and captured using a Coolsnap EZ camera (Photometrics) through MetaVue Software (Molecular Devices)*.* Specific band pass filter sets for DAPI, FITC and Texas Red were used for visualizing DAPI, GFP and phalloidin-Atto590, respectively. Images were processed and analysed using ImageJ (http://rsb.info.nih.gov/ij).

## Results

### Design of novel *C. jejuni* chromosomal expression systems

**Fig. 1:**
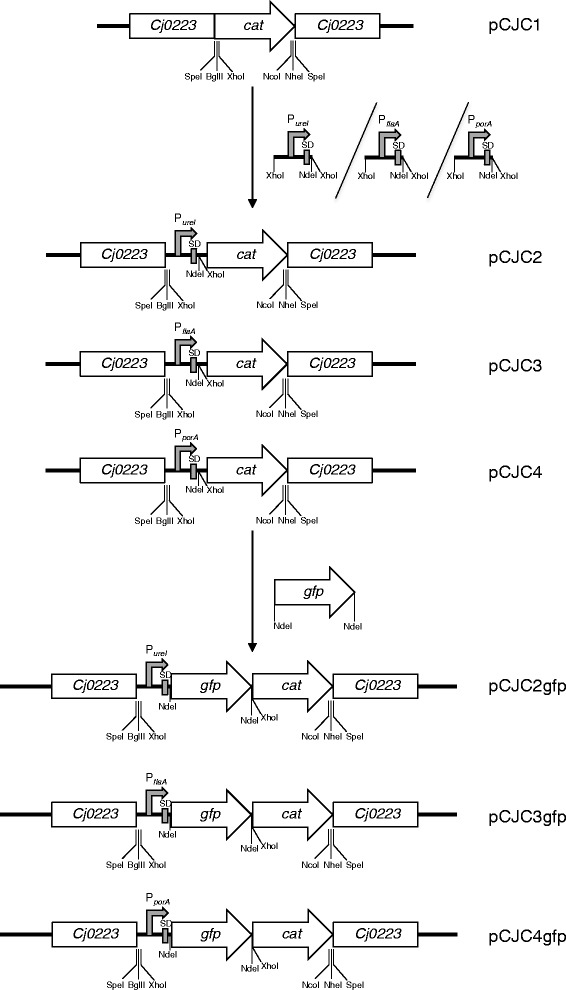
Construction of expression vectors for integration onto the *C. jejuni* chromosome. Construction of the pCJC series of plasmids for integration in the *C. jejuni* 11168 pseudogene Cj0223. A fragment of Cj0223 cloned into pUC18 is interrupted by a chloramphenicol resistance cassette (*cat*) at a unique SpeI site. Promoter regions with Shine-Dalgarno (SD) sites were cloned into the XhoI site followed by insertion of *gfp* at the NdeI site. Three promoter regions were used: *H. pylori ureI* to create pCJC2, *H. pullorum flaA* to create pCJC3 and *C. jejuni porA* to create pCJC4

**Fig. 2:**
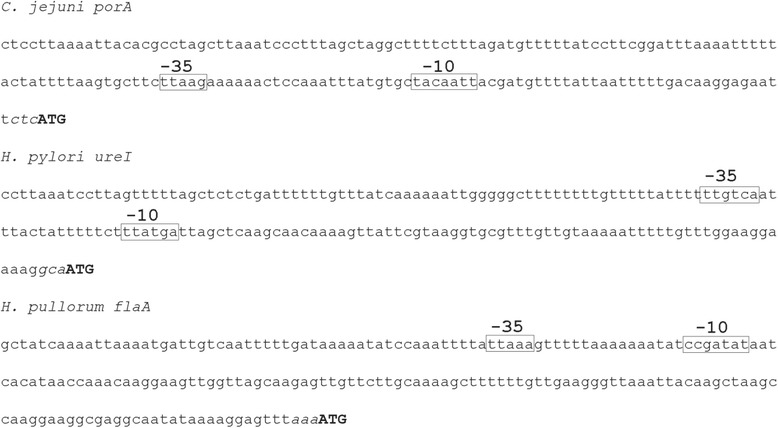
Promoter regions employed in this study. The *H. pylori* 26695 *ureI* promoter region is previously characterised, the *C. jejuni* 11168 *porA* and *H. pullorum* NCTC 12824 *flaA* promoters are putative based on known consensus sequences. Boxed sequences denote known or putative −10 and −35 regions with the start codon of the associated open reading frames in bold capitals. Sequences were amplified by PCR to include 5′ and 3′ restriction sites and italicised bases were altered to “CAT” to create an NdeI site at the 3′ end

We have previously used a vector for genetic complementation in *C. jejuni* consisting of a 2179 bp fragment (corresponding to nt 205297–207475 inclusive of the *C. jejuni* NCTC 11168 genome sequence) cloned into pUC18 with a chloramphenicol resistance (*cat)* cassette cloned into a central SpeI site [11]. The *cat* gene is thus flanked by regions from pseudogene Cj0223, so that electroporation of this vector into *C. jejuni* cells results in integration, through a double recombination event, of the *cat* cassette and gene-of-interest onto the chromosome within Cj0223. This vector was modified by introduction of BglII/XhoI and NcoI/NheI sites flanking the *cat* cassette to produce plasmid pDENNIS [16]. This facilitated insertion of genes either upstream or downstream of *cat* and was used successfully for genetic complementation in *C. jejuni* [16]. We have now renamed pDENNIS as pCJC1 (Fig. 1). To further develop this vector providing increased and more flexible expression levels of genes introduced onto the *C. jejuni* chromosome, we have modified pCJC1 by cloning three distinct promoter regions, including native Shine-Dalgarno (SD) sequences and associated start codons (Fig. 2) immediately upstream of the *cat* gene (Fig. 1). Promoter regions (described below) were selected from *Campylobacter* and *Helicobacter* species based on previous evidence of their relatively high activity.

The first, (P_*porA*_), is from the *C. jejuni* NCTC 11168 *porA* gene. Under several different growth conditions *porA* transcript is one of the most abundant in the transcriptome [17, 18]. Although transcription from P_*porA*_ has been studied [19, 20], the promoter has not been mapped in detail. Our *in silico* analysis of the *porA* upstream region identified a putative SD site and a σ^70^-type promoter approximately 170 bp upstream of the start codon (Fig. 2). The second σ^70^-type promoter region chosen, P_*ureI*_ from the *H. pylori ureI* gene (Fig. 2), was previously used in a plasmid-based inducible expression system for *H. pylori* [21]. The third promoter (P_*flaA*_) is from the *Helicobacter pullorum* NCTC 12824 *flaA* gene (Fig. 2), a species studied in our laboratory. The *flaA* σ^28^-dependent promoters from *C. jejuni* [22] and *H. pylori* [23] are well characterized. A σ^28^ type promoter and SD site were identified upstream of *H. pullorum flaA* although the putative −10 and −35 regions are significantly further upstream (154 bp) compared to those from *C. jejuni* (54 bp) and *H. pylori* (57 bp) (Additional file 1: Figure S1).

Each promoter region with cognate SD site and ATG start site was PCR amplified to include 5′ and 3′ XhoI restriction sites allowing insertion into the unique XhoI site upstream of the pCJC1 *cat* cassette (Fig. 1). Primers were designed so that an NdeI site was placed adjacent to the XhoI site located immediately upstream of the *cat* cassette (Fig. 1). The NdeI recognition sequence of CA*TATG and primer design was such that the three bases preceding the start codon were altered to CAT to create the NdeI site (Fig. 2). This potentially allows introduction of a gene at the NdeI site creating a translational fusion to the start codon without altering the spacing between SD and start codon. Plasmids constructed in this way containing the P_*ureI*_, P_*flaA*_ and P_*porA*_ promoters were named pCJC2, pCJC3 and pCJC4 respectively (Fig. 1).

### Analysis of relative expression levels in *Campylobacter jejuni* 11168

To test the relative expression levels from each of the promoter regions in *C. jejuni* we commercially synthesised a *C. jejuni* codon-optimised version of the *gfp* + gene [13, 23] and this was named *gfp*^*Cj*^ (Additional file 2: Figure S2, GenBank accession KP994992). The *gfp*^*Cj*^ gene was PCR amplified with 5′ and 3′ NdeI sites using primers gfp-F and gfp-R (Table 1) and inserted into pCJC2, pCJC3 and pCJC4 in the same transcriptional orientation as the *cat* gene, creating plasmids pCJC2gfp^*Cj*^, pCJC3gfp^*Cj*^ and pCJC4*gfp*^*Cj*^ respectively (Fig. 1). These *C. jejuni* suicide vectors were electroporated into strain 11168 and transformants identified in which a double recombination event had occurred at the Cj0223 locus. Strains constructed in this way were termed 11168gfp2, 11168gfp3 and 11168gfp4 and no significant effect on growth compared to the parental strain was observed (data not shown). To investigate promoter strength, relative fluorescence of early/mid-exponential phase cells of *C. jejuni* NCTC 11168 and the three *gfp*^*Cj*^ expressing strains was measured in a 96-well plate reader (see Methods).

**Fig. 3:**
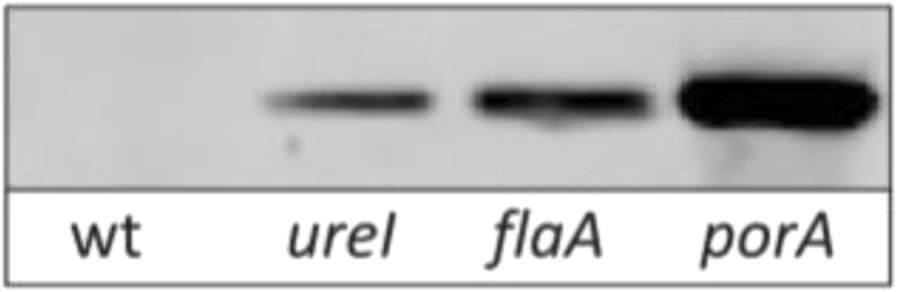
GFP levels from single copy chromosomal *gfp* ^*Cj*^ in *C. jejuni* strains 11168gfp2, 11168gfp3 and 11168gfp4. GFP detected by Western blotting in whole-cell lysates from strains 11168gfp2 (*ureI*), 11168gfp3 (*flaA*) and 11168gfp4 (*porA*)

All three strains (11168gfp2, 11168gfp3 and 11168gfp4) displayed significantly higher fluorescence than wild-type 11168 cells. The levels of fluorescence from 11168gfp2, 11168gfp3 and 11168gfp4 were 106.0 (+/− 2.1 SE), 197.6 (+/− 23.1 SE) and 4002.9 (+/− 252.5 SE) arbitrary fluorescence units /OD_600_ respectively reflecting activity of their corresponding promoters P_*ureI*_, P_*flaA*_ and P_*porA*_. Parallel Western blotting experiments of standardized whole-cell lysates from 11168gfp2, 11168gfp3 and 11168gfp4 using an anti-GFP antibody confirmed the *gfp* expression level pattern of P_*ureI*_ < P_*flaA*_ < P_*porA*_ (Fig. 3). The varying expression levels of these three promoter regions will allow a choice of promoter for recombinant gene expression in *C. jejuni* based upon the desired application.

**Fig. 4:**
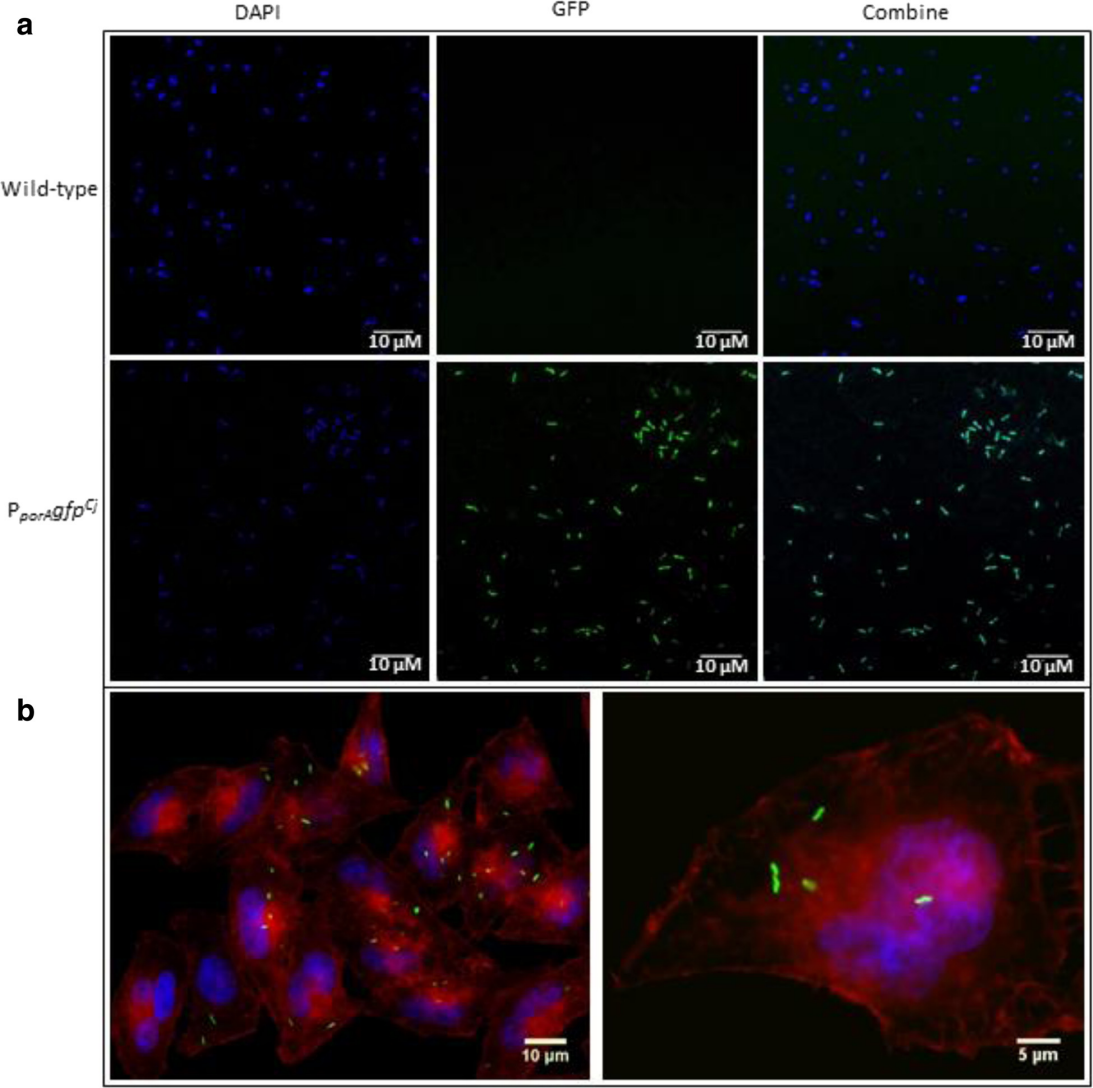
Fluorescent microscopy of *C. jejuni* 11168gfp4 cells. **a**. Fluorescent imaging of cells of wild-type *C. jejuni* NCTC 11168 and the 11168gfp4 strain expressing chromosomal *gfp* ^*Cj*^ driven by the *C. jejuni* promoter P_*porA*_. **b**. Fluorescent imaging of gentamicin-treated HeLa cells, 3 h post-infection with *C. jejuni* NCTC 11168gfp4 expressing chromosomal *gfp* ^*Cj*^ driven by the *C. jejuni* promoter P_*porA*_. Cells were stained with DAPI (blue) and phalloidin (red) with GFP fluorescence in green

Cultures of the *C. jejuni* 11168gfp4 strain with the P_porA_ promoter driving expression of *gfp*^*Cj*^ displayed very high fluorescence levels and so we tested its utility for in vitro imaging. Broth-grown cultures of 11168gfp4 were stained with DAPI and visualised for DAPI and GFP fluorescence by standard fluorescence microscopy (see Methods). Cells were clearly visible by GFP fluorescence (Fig. 4a) and fluorescence levels appeared consistent across individual cells as judged by comparison with DAPI staining, indicating homogenous levels of GFP production in the population, important for in vitro and in vivo experiments. We therefore used strain *C. jejuni* 11168gfp4 to infect HeLa cells in a basic cell invasion assay to determine if fluorescence levels were sufficient to monitor intracellular *C. jejuni*. Mid-log phase *C. jejuni* grown in broth were used to infect 24 h HeLa cells with an MOI of 10 prior to treatment with gentamicin to kill extracellular bacteria. Fixed HeLa cells were viewed by fluorescence microscopy following staining of cellular actin. Highly fluorescent intracellular *C. jejuni* cells were readily visualized within the actin and DAPI-stained HeLa cells (Fig. 4b). These observations confirm the utility of the combination of high activity promoter with insertion onto the *C. jejuni* chromosome to produce strains that stably express genes at high level. .

## Discussion

Genetic tools for the expression of recombinant genes are important for a number of applications and the lack of such tools for *C. jejuni* has been a limiting factor in experimental design. Here we have described a flexible set of constructs for the stable expression of genes at relatively low, medium and very high levels after chromosomal integration downstream of three distinct promoters. These expression levels can be used for applications such as functional complementation when low/medium expression levels might be desired and for the production and purification of specific proteins employing higher expression levels. The utility of very high-level expression from the P_*porA*_ promoter was demonstrated by creating the highly fluorescent *C. jejuni* 11168gfp4 strain. The constructs described (pCJCgfp2, pCJCgfp3 and pCJCgfp4) could also be used to produce *gfp* translational fusions for determining the cellular localisation of specific proteins.

We have used a method for integrating genes onto the *C. jejuni* chromosome that targets pseudogene Cj0223 to minimize potential for a deleterious effect on cells. Cj0223 contains multiple frame-shift mutations/in-frame stop codons and is therefore non-functional and has been previously used to insert genes onto the *C. jejuni* chromosome [11]. The Cj0223 pseudogene is also present in most commonly used strains of *C. jejuni* so that this approach should be generally applicable.

The promoter elements used in this study were chosen based on previous studies describing either their activity or their utility in similar expression systems. The *C. jejuni porA* promoter is one of the strongest constitutive promoters in recent RNA-seq transcriptome studies, and PorA is the dominant protein in SDS-PAGE of *C. jejuni* whole-cell lysates [17, 18]. Promoter P_*flaA*_ from *H.pullorum* was used as high levels of FlaA protein are produced in both *C. jejuni* and *H. pylori*. Promoter P_*ureI*_ from *H. pylori* is a constitutive promoter successfully used in a *H. pylori* plasmid-based inducible expression system [21, 24]. To measure activity of these promoters we employed GFP as a reporter. Codon optimization of *gfp* for expression in *C. jejuni* more than doubled fluorescence observed with the P_*porA*_ promoter (data not shown) highlighting the importance of considering specific translational features of host background. Indeed this *gfp*^*Cj*^ allele might be considered for more general use in *C. jejuni* for applications such as a reporter gene for transcriptional and translational analysis and as a fusion partner for protein localization and quantitation. Comparison of fluorescence levels for strains expressing *gfp* from P_*ureI*_, P_*flaA*_ and P_*porA*_ demonstrated an approximately doubling of fluorescence with P_*flaA*_ compared to P_*ureI*_ and a further approximately twenty-fold increase with P_*porA*_. We propose that P_*porA*_ should be the promoter of choice when high-level expression of recombinant proteins is desired although potential toxicity issues should be considered. The high level expression from P_*porA*_ is also very useful for protein purification. High level expression of genes to produce specific proteins for structural/functional characterisation is generally performed in *E. coli* as this allows rapid and inexpensive production of large amounts of biomass and hence protein. However in certain circumstances where proteins may be post-translationally modified, for example by the protein glycosylation systems found in Campylobacters and some Helicobacter species, then production in these particular species is required to produce appropriately modified proteins. We successfully used promoter P_*porA*_ to drive expression of *hgpA* encoding an *N*-linked glycoprotein in *H. pullorum* with sufficient yields for purification of milligrams of HgpA glycoprotein (data not shown).

In summary, the vectors presented in this work provide a useful set of tools to aid in molecular studies of this important bacterial pathogen and the principles involved in designing these tools should be generally applicable to other bacteria. Additionally we have constructed a new *gfp* allele that in combination with the expression systems described produced a highly fluorescent *C. jejuni* strain expressing *gfp*^Cj^ from the chromosome for potential use in both in vitro and in vivo infection studies. The advent of synthetic biology and the affordable technology of gene synthesis will facilitate further development of these and other systems to produce a valuable genetic toolbox to aid in *Campylobacter* and *Helicobacter* research.

## Conclusions

We have constructed vectors allowing recombination-mediated incorporation of genes onto the *C. jejuni* chromosome downstream of one of three promoters with varying expression levels. These vectors will be useful for genetic complementation, expression of genes at relatively high levels and construction of GFP translational fusions in this important bacterial pathogen.

## Additional files

Additional file 1: Figure S1. Sigma 28-dependent promoters of *flaA* genes from *C. jejuni* NCTC 11168, *H. pylori* 26695 and *H. pullorum* NCTC 12824. *C. jejuni* and *H. pylori* promoters are characterized [22, 23] and the putative promoter for *H. pullorum* is uncharacterized*.* Boxed sequences denote -10 and -35 regions, text in bold uppercase the *flaA* open reading frame start codon, text in bold the transcriptional start site and text in bold and italics the Shine-Dalgarno sequences. (DOCX 135 kb) Additional file 2: Figure S2. Nucleotide sequence of the *C. jejuni* codon-optimised *gfp* gene (*gfp* ^*Cj*^). (DOCX 19 kb)

## Abbreviations

*cat*: Chloramphenicol resistance cassette
DAPI: 4′,6-diamidino-2-phenylindole
DMEM: Dulbecco’s Modified Eagles Medium
GFP: Green fluorescent protein
LB: Luria Bertani
MOI: Multiplicity of infection
PBS: Phosphate buffered saline
PBSD: Dulbecco’s phosphate buffered saline
