# Supplementary material for: Chromosomal integration vectors allowing flexible expression of foreign genes in Campylobacter jejuni
Source: BMC Microbiol. 2015 Oct 24;15:230. doi: 10.1186/s12866-015-0559-5 (PMC4619491; doi:10.1186/s12866-015-0559-5)
Supplement: Additional file 1: Figure S1. — Sigma 28-dependent promoters of flaA genes from C. jejuni NCTC 11168, H. pylori 26695 and H. pullorum NCTC 12824. C. jejuni and H. pylori promoters are characterized [22, 23] and the putative promoter for H. pullorum is uncharacterized. Boxed sequences denote -10 and -35 regions, text in bold uppercase the flaA open reading frame start codon, text in bold the transcriptional start site and text in bold and italics the Shine-Dalgarno sequences. (DOCX 135 kb) [file 12866_2015_559_MOESM1_ESM.docx]

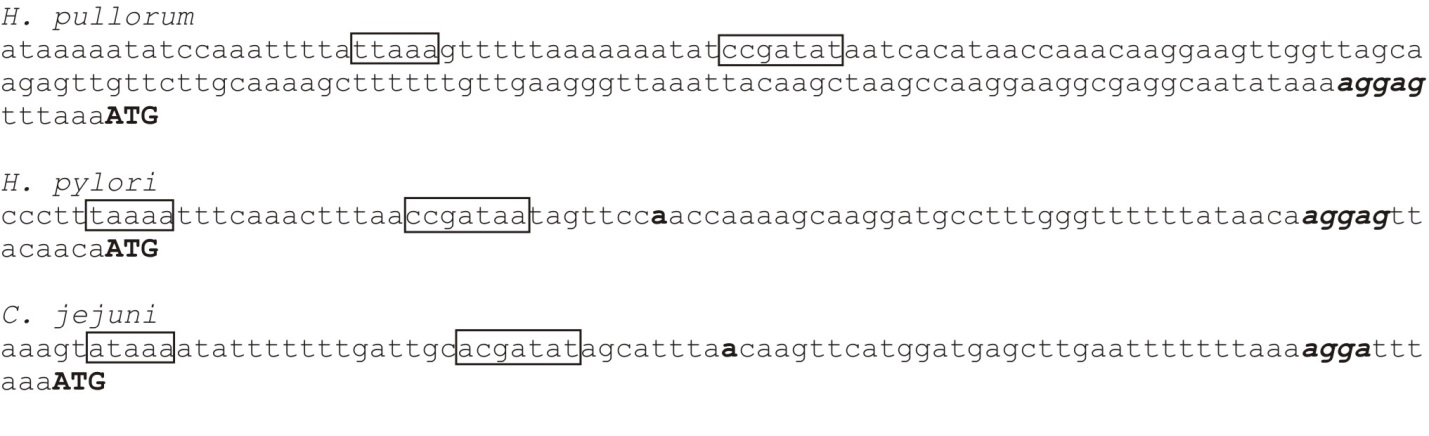


**Figure S1. Sigma 28-dependent promoters of *flaA* genes from *C. jejuni* NCTC 11168, *H. pylori* 26695 and *H. pullorum* NCTC 12824.** *C. jejuni* and *H. pylori* promoters are characterized [22, 23] and the putative promoter for *H. pullorum* is uncharacterized*.* Boxed sequences denote -10 and -35 regions, text in bold uppercase the *flaA* open reading frame start codon, text in bold the transcriptional start site and text in bold and italics the Shine-Dalgarno sequences.
