# Supplementary material for: Chromosomal integration vectors allowing flexible expression of foreign genes in Campylobacter jejuni
Source: BMC Microbiol. 2015 Oct 24;15:230. doi: 10.1186/s12866-015-0559-5 (PMC4619491; doi:10.1186/s12866-015-0559-5)
Supplement: Additional file 2: Figure S2. — Nucleotide sequence of the C. jejuni codon-optimised gfp gene (gfp Cj). (DOCX 19 kb) [file 12866_2015_559_MOESM2_ESM.docx]

>gfpCj

1 ATGAGTAAAG GTGAAGAATT ATTTACTGGT GTTGTTCCTA TTTTAGTTGA ATTAGATGGT

61 GATGTTAATG GTCATAAATT TAGTGTTAGT GGTGAAGGTG AAGGTGATGC TACTTATGGT

121 AAATTAACTT TAAAATTTAT TTGTACTACT GGTAAATTAC CTGTTCCTTG GCCTACTTTA

181 GTTACTACTT TAACTTATGG TGTTCAATGT TTTAGTAGAT ATCCTGATCA CATGAAAAGA

241 CATGATTTTT TTAAAAGTGC TATGCCTGAA GGTTATGTTC AAGAAAGAAC TATTAGTTTT

301 AAAGATGATG GTAATTATAA AACTAGAGCT GAAGTTAAAT TTGAAGGTGA TACTTTAGTT

361 AATAGAATTG AATTAAAAGG TATTGATTTT AAAGAAGATG GTAATATTTT AGGTCATAAA

421 TTAGAATATA ATTATAATAG TCATAATGTT TATATTACTG CTGATAAACA AAAAAATGGT

481 ATTAAAGCTA ATTTTAAAAT TAGACATAAT ATTGAAGATG GTAGTGTTCA ATTAGCTGAT

541 CATTATCAAC AAAATACTCC TATTGGTGAT GGTCCTGTTT TATTACCTGA TAATCATTAT

601 TTAAGTACTC AAAGTGCTTT AAGTAAAGAT CCTAATGAAA AAAGAGATCA CATGGTTTTA

661 TTAGAATTTG TTACTGCTGC TGGTATTACT CATGGTATGG ATGAATTATA TAAATAA

**Figure S2. Nucleotide sequence of the *C. jejuni* codon-optimised *gfp* gene (*gfp^Cj^*).**
